# Phenotypic Characterization of Immortalized Chondrocytes from a Desbuquois Dysplasia Type 1 Mouse Model: A Tool for Studying Defects in Glycosaminoglycan Biosynthesis

**DOI:** 10.3390/ijms22179304

**Published:** 2021-08-27

**Authors:** Chiara Gramegna Tota, Beatrice Valenti, Antonella Forlino, Antonio Rossi, Chiara Paganini

**Affiliations:** Department of Molecular Medicine, Unit of Biochemistry, University of Pavia, 27100 Pavia, Italy; chiara.gramegnatota01@universitadipavia.it (C.G.T.); beatrice.valenti@cro.it (B.V.); antonella.forlino@unipv.it (A.F.); chiara.paganini@unipv.it (C.P.)

**Keywords:** proteoglycan, collagen, cartilage, extracellular matrix, in vitro model, immortalization, skeletal dysplasia

## Abstract

The complexity of skeletal pathologies makes use of in vivo models essential to elucidate the pathogenesis of the diseases; nevertheless, chondrocyte and osteoblast cell lines provide relevant information on the underlying disease mechanisms. Due to the limitations of primary chondrocytes, immortalized cells represent a unique tool to overcome this problem since they grow very easily for several passages. However, in the immortalization procedure the cells might lose the original phenotype; thus, these cell lines should be deeply characterized before their use. We immortalized primary chondrocytes from a *Cant1* knock-out mouse, an animal model of Desbuquois dysplasia type 1, with a plasmid expressing the SV40 large and small T antigen. This cell line, based on morphological and biochemical parameters, showed preservation of the chondrocyte phenotype. In addition reduced proteoglycan synthesis and oversulfation of glycosaminoglycan chains were demonstrated, as already observed in primary chondrocytes from the *Cant1* knock-out mouse. In conclusion, immortalized *Cant1* knock-out chondrocytes maintained the disease phenotype observed in primary cells validating the in vitro model and providing an additional tool to further study the proteoglycan biosynthesis defect. The same approach might be extended to other cartilage disorders.

## 1. Introduction

Chondrocyte is the unique cell type in cartilage and is essential for cartilage formation and function. These cells are embedded in an extracellular matrix (ECM) consisting of complexes of aggrecan, hyaluronan, and link protein in a collagen fibril network [1]. In articular cartilage, the ECM synthesized by chondrocytes imparts to the tissue stiffness, wear resistance, and the ability to withstand compressive forces, allowing the joints to move smoothly and without pain [2]. The other major function of chondrocytes occurs in endochondral ossification, in which chondrocytes go through a process of maturation characterized by type X collagen expression, loss of aggrecan and type II collagen expression and cellular hypertrophy, leading to the formation of many skeletal elements and to longitudinal growth of long bones [3].

Chondrocytes maintain cartilage homeostasis by balanced anabolic and catabolic activities. This equilibrium is disrupted in osteoarthritis, the major degenerative pathology associated with chondrocytes. Osteoarthritis is characterized by the increased expression of ECM degrading enzymes resulting in cartilage destruction by degradation of the collagen and proteoglycan (PG) moieties [4]. Cartilage development and homeostasis is also altered in several genetic diseases that affect the skeleton. The updated nosology comprises 461 different disorders that range in severity from mild to severe and lethal forms; the gene defects have been characterized in 92% of these human syndromes [5].

Different in vivo and in vitro models have been developed in order to approach the complex biology of cartilage in normal and pathological conditions. Analysis of genetically modified mice provides a powerful method to study the role of gene products in vivo. Mouse models of osteoarthritis as well as of several skeletal dysplasias have been used to elucidate the effect of a single defect on the disease pathogenesis [6,7,8]. However, in vitro studies are a powerful tool to investigate specific aspects of cartilage biology that cannot be studied in the whole animal due to its complexity. Isolated primary chondrocytes present phenotype instability with increased time in culture providing an important limitation to chondrocyte use. The expression levels of major chondrocyte markers (i.e., type II and type IX collagen, COMP) significantly decrease with culture passages suggesting loss of the chondrocyte phenotype [9,10]. Thus, one of the main challenges to establish chondrocyte cell lines is to maintain the chondrocyte phenotype. To this purpose, high density aggregate culture methods [11] or co-cultures of chondrocytes with mesenchymal stem cells [12] have been described. In addition, approaches to re-differentiation of de-differentiated chondrocytes have been investigated in 3D culture using different gels containing alginate [13], agarose [14], hyaluronan [15], and a combination of collagen and alginate [16]. These culture systems maintain the chondrocyte phenotype, but since cells do not proliferate, they do not overcome the limitation in cell supply. Thus, investigators have generated immortalized chondrocyte cell lines from human controls and wild-type mice with different methods to preserve the phenotype and to provide a surrogate model system to study cartilage biology [17,18,19,20,21,22,23,24,25,26,27]. Most of these cell lines have been generated to study specific anabolic and catabolic pathways in order to provide additional information on the molecular mechanism of osteoarthritis development and progression [18,26]. To investigate the involvement of particular gene products in pathological mechanisms, these immortalized cells might be further modified by knocking down specific genes through RNA interference or gene editing. However, this approach, which have been reported in several cell lines such as ATDC5 [28] and Swarm rat chondrosarcoma cells [29], requires further subculturing to attain genetically modified clonal lines and, in addition, carries the risk of off-target effects. These problems can be overcome by the immortalization of chondrocytes from mouse models of genetic disorders of the skeleton to study putative disease pathways and/or ECM protein synthesis and assembly in simple and well-defined in vitro systems. Structural and biosynthetic defects in cartilage ECM proteins, including collagens, PGs, and glycoproteins (COMP, matrilin-3) result in several dominant and recessive skeletal dysplasias. Moreover, due to the relevance of the oligosaccharide part of PGs for their biological functions, different forms of skeletal disorders are caused by defects in glycosaminoglycan (GAG) biosynthesis, a complex process that involves several transporters and enzymes, most of them localized in the Golgi apparatus [30,31]. Thus, the use of in vitro systems [32] is ideal for a deep characterization of the biosynthetic defects. 

We recently generated a *Cant1* knock-out mouse for the human Desbuquois dysplasia type 1 (DBQD1) [33]. This autosomal recessive chondrodysplasia is characterized by short stature, joint laxity, short extremities, typical hand anomalies, and progressive scoliosis. DBQD1 is caused by mutations in the calcium activated nucleotidase 1 (*CANT1*) gene encoding for an ER/Golgi nucleotidase that hydrolyses UDP [34,35]. We demonstrated that its impairment causes reduced synthesis, oversulfation, and reduced hydrodynamic size of GAGs [33], suggesting that CANT1 affects the whole biosynthetic process of GAGs occurring in the Golgi. For this reason, in vitro tools are necessary to dissect the complex pathway of GAG biosynthesis. 

In this work we generated and characterized immortalized cell lines from a *Cant1* knock-out mouse [33]. These cell lines showed the defects in GAG biosynthesis already observed in primary chondrocytes from the same mouse model and they provide an in vitro tool for modeling the molecular mechanisms of skeletal defects caused by *Cant1* impairment.

## 2. Results

### 2.1. Immortalization of Murine Chondrocytes from Femoral Head Cartilage

**Figure 1:**
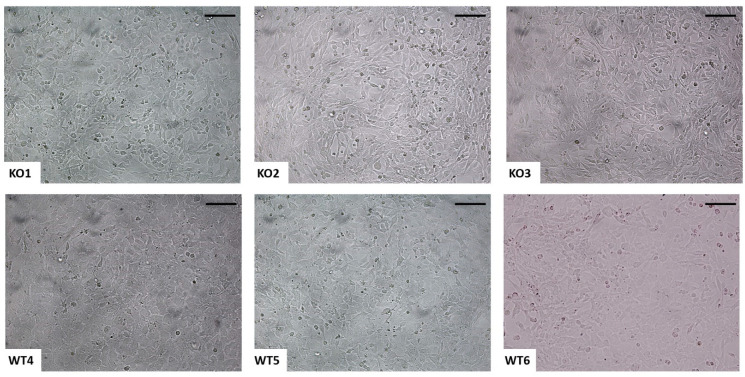
Morphology of immortalized chondrocytes. Representative images of immortalized cell lines at early confluence. Both *Cant1* knock-out (KO1, KO2, and KO3) and wild-type (WT4, WT5, and WT6) cells maintained the typical polygonal chondrocyte morphology after immortalization. Scale bar 100 µm.

Primary chondrocytes isolated from femoral head cartilage of *Cant1* knock-out and wild-type mice were transfected with the plasmid vector pZipSV766-1 expressing the large and small T antigen of SV40 and containing the neomycin resistance gene. Two days after transfection, neomycin resistant cells were selected by treatment with G418. The cells were passaged at high dilution and three colonies for each genotype were isolated. Cells from the selected clones showed a polygonal morphology characteristic of chondrocytes rather than fibroblasts (Figure 1), suggesting that cells maintained the chondrocyte phenotype. Thus, these cell lines were further characterized at the molecular level.

### 2.2. Analysis of the Cartilage Phenotype in Immortalized Chondrocytes

**Figure 2:**
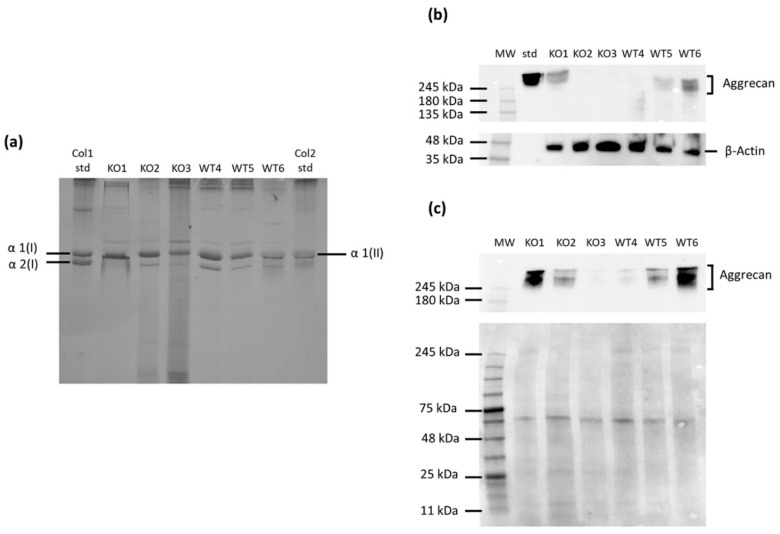
Synthesis of extracellular matrix proteins in immortalized chondrocytes. (**a**) 6% SDS-PAGE of collagen purified by pepsin digestion from the medium showed that all cell lines synthesized type II collagen, but a very small amount of type I collagen was present in wild-types and two *Cant1* knock-out samples. Col1 std = pepsin solubilized type I collagen standard and Col2 std = pepsin solubilized type II collagen standard. (**b**) Western blot of proteins extracted from cell layer of immortalized chondrocytes was performed with specific antibodies against aggrecan core protein and against β-Actin as internal control. The blot is representative of three independent experiments. MW = molecular weight and std = aggrecan core standard. (**c**) Western blot of proteins extracted from the medium was performed with a specific antibody against the aggrecan core protein; protein stain of the PVDF membrane with swift membrane stain was used as loading control. The blot is representative of two independent experiments. MW = molecular weight. Both cell layer and medium Western blots showed huge variability of aggrecan synthesis among immortalized chondrocytes; KO1 and WT6 were the *Cant1* knock-out and wild-type samples, respectively, that synthesized more aggrecan. The full original gel and Western blots are reported in Supplementary Materials Figure S1.

We further characterized the phenotype of immortalized cell lines analyzing the expression level of the main ECM proteins synthesized by chondrocytes: type II collagen and aggrecan. It is well known that chondrocytes shift to a fibroblast-like phenotype when cultured in monolayer after several passages [9]. Since type II collagen is the typical differentiation marker of the chondrocyte phenotype, collagens secreted in the culture medium by immortalized cells were purified by pepsin digestion and analyzed by 6% SDS-PAGE (Figure 2a). Coomassie staining of the gel showed a single band in the type II collagen standard, corresponding to the α1 chain of type II collagen (α1(II)), and two bands in the standard of type I collagen, corresponding to the α1 and α2 chains of type I collagen (α1(I) and α2(I), respectively). Since the α1 chains of type I and type II collagen comigrate, the absence of the α2(I) band indicated that the α1 band consisted primarily of type II collagen. In all cell lines, both wild-type and knock-out, the α1(II) band was present indicating that the cells synthesized mainly type II collagen maintaining the chondrocyte phenotype. However, in several cell lines, both wild-type and knock-out, a faint α2(I) band was observed demonstrating that these cell lines synthesized very low levels of type I collagen. Among knock-out samples, the α2(I) band was not detected at all in KO1, while in WT6 the α2(I) band was negligible compared with the other wild-type cell lines (Figure 2a) and quantitated as less than 2% of the α1(I+II) collagen band by densitometric scanning of stained gels. This observation suggested that KO1 and WT6 were the knock-out and wild-type cell lines, respectively, that better preserved the chondrocyte phenotype. 

Immortalized cell lines were also screened for the expression of aggrecan, the main PG present in cartilage. For this purpose, aggrecan in the culture medium and the cell layer was analyzed by Western blot with a specific aggrecan antibody. An equal amount of proteins was loaded on gel for cell layer samples, while an equal volume was loaded on the gel for the culture medium. Western blot analysis showed a marked heterogeneous expression of aggrecan (Figure 2b,c). Among the different immortalized knock-out and wild-type cell lines, expression of this PG was quite evident in KO1 and WT6, respectively. This result confirmed that KO1 and WT6 cell lines had preserved the chondrocyte phenotype as already demonstrated by type II collagen analysis.

**Figure 3:**
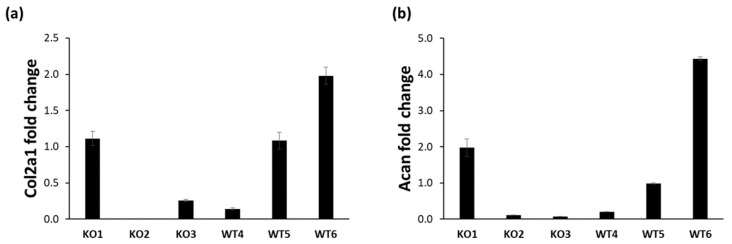
Expression of extracellular matrix proteins in immortalized chondrocytes. (**a**) The type II collagen (Col2a1) expression was assessed by real-time PCR. Fold change was calculated by ΔΔCt method using Gapdh as housekeeping gene and WT5 as reference sample random selected. Data are reported as mean ± standard deviation of three replicates. (**b**) The aggrecan (Acan) expression was assessed by real-time PCR. Fold change was calculated by ΔΔCt method using Gapdh as housekeeping gene and WT5 as reference sample random selected. Data are reported as mean ± standard deviation of three replicates. All samples expressed type II collagen and aggrecan, even if with a huge variability among cell lines.

Type II collagen and aggrecan expression was also studied at the mRNA level by real-time PCR. The results confirmed that all immortalized cell lines expressed type II collagen and aggrecan, even if with huge variability (Figure 3). Among immortalized knock-out chondrocytes the highest type II collagen and aggrecan expression was in KO1 cell line, while in wild-type cell lines WT6 showed the highest expression level of type II collagen and aggrecan. 

Overall, these results demonstrated that the cell lines KO1 and WT6, obtained by the immortalization of *Cant1* knock-out and wild-type chondrocytes, respectively, maintained the chondrocyte phenotype based on expression at the protein and mRNA level of the two main cartilage ECM proteins. These cell lines were used to further investigate the cell phenotype at the biochemical level.

### 2.3. Glycosaminoglycan Synthesis and Sulfation in Immortalized Cant1 Knock-Out and Wild-Type Chondrocytes

In a previous work, we demonstrated that *Cant1* knock-out chondrocytes in primary culture synthesize reduced amount of GAGs which, in addition, were oversulfated [33]. 

**Figure 4:**
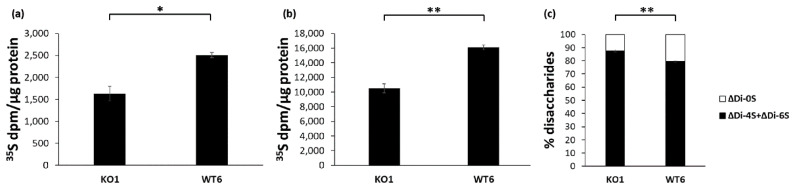
*Cant1* knock-out chondrocytes maintained the mutant phenotype after immortalization. (**a**) Immortalized chondrocytes were metabolically labeled with ^35^S-sulfate for 24 h in basal medium. The amount of newly synthesized GAGs was determined normalizing the ^35^S-activity to the protein content. Data are reported as mean ± standard deviation of three experiments. Significance between KO1 and WT6 samples was analyzed by Student’s *t*-test; * *p* < 0.05. (**b**) Immortalized chondrocytes were metabolically labeled with ^35^S-sulfate for 24 h in basal medium containing 1 mM *p*-nitrophenyl-β-D-xylopyranoside. The amount of newly synthesized GAGs was determined normalizing the ^35^S-activity to the protein content. Data are reported as mean ± standard deviation of three experiments. Significance between KO1 and WT6 samples was analyzed by Student’s *t*-test; ** *p* < 0.01. In both condition, basal medium with and without *p*-nitrophenyl-β-d-xylopyranoside, immortalized KO1 chondrocytes synthesized less GAGs compared with immortalized WT6 cells as demonstrated previously in primary chondrocytes [33]. (**c**) Sulfation of chondroitin sulfate PGs extracted from medium of immortalized chondrocytes was analyzed by HPLC after digestion with chondroitinases ABC and ACII. Data are reported as mean ± standard deviation of three replicates. Significance between KO1 and WT6 samples was analyzed by Student’s *t*-test; ** *p* < 0.01. Immortalized KO1 chondrocytes showed a higher percentage of monosulfated disaccharides (ΔDi-4S + ΔDi-6S) compared with immortalized WT6 cells as demonstrated previously in primary chondrocytes [33].

PG synthesis in immortalized *Cant1* knock-out (KO1 cell line) and wild-type (WT6 cell line) chondrocytes was studied by metabolic labeling with ^35^S-sulfate for 24 h. At the end of the labeling period, macromolecules were purified by ion exchange chromatography and GAGs were quantified by measuring the ^35^S-activity and normalized to the protein content. In basal conditions, GAG synthesis was reduced in immortalized KO1 chondrocytes compared with immortalized WT6 cells (1629 ± 167 dpm/µg protein vs. 2505 ± 60 dpm/µg protein, respectively; *p* = 0.020) (Figure 4a). GAG synthesis was further studied by metabolic labeling in medium containing *p*-nitrophenyl β-D-xylopyranoside, a molecule that enhances GAG synthesis. In both cell lines, GAG synthesis was markedly increased compared to the basal condition. Moreover, GAG synthesis was significantly higher in WT6 chondrocytes compared with KO1 cells (16,116 ± 312 dpm/µg protein vs. 10,509 ± 596 dpm/µg protein, respectively; *p* = 0.007) (Figure 4b) as observed in the basal condition. 

GAG synthesis was further characterized by sulfation analysis of chondroitin sulfate PGs secreted in the culture medium of immortalized chondrocytes. Purified GAGs were digested with chondroitinase ABC and ACII and released chondroitin sulfate disaccharides analyzed by HPLC after fluorescence derivatization. The percentage of monosulfated disaccharides (ΔDi-4S and ΔDi-6S) relative to the total amount of disaccharides (ΔDi-0S, ΔDi-4S, and ΔDi-6S) was increased in KO1 cells compared with WT6 ones (87.57 ± 0.82% vs. 79.42 ± 0.61% monosulfated disaccharides, respectively; *p* = 0.008) demonstrating GAG oversulfation in the *Cant1* knock-out cell line compared to the wild-type one (Figure 4c). 

In conclusion, the GAG synthesis and sulfation defects demonstrated in immortalized *Cant1* knock-out chondrocytes mimicked the defects previously described in primary cells from the same mouse model of DBQD1 [33].

### 2.4. Characterization of the Extracellular Matrix Produced by Immortalized Cells

One of the main features of chondrocytes is their ability to synthesize a huge amount of ECM. To analyze the synthesis and deposition of the ECM by immortalized *Cant1* knock-out and wild-type chondrocytes, KO1, and WT6 cells were incubated for 12 days in DMEM containing 5% FBS in presence or absence of ITS. Insulin, contained in this serum substitute (as in Nutridoma-SP) together with transferrin and sodium selenite, promotes chondrogenesis in ATDC5 cells and favors the differentiated phenotype in immortalized human chondrocytes [21,23,28]. The ECM produced over 12 days was analyzed by Alcian blue staining, aggrecan Western blot, and immunofluorescence. 

**Figure 5:**
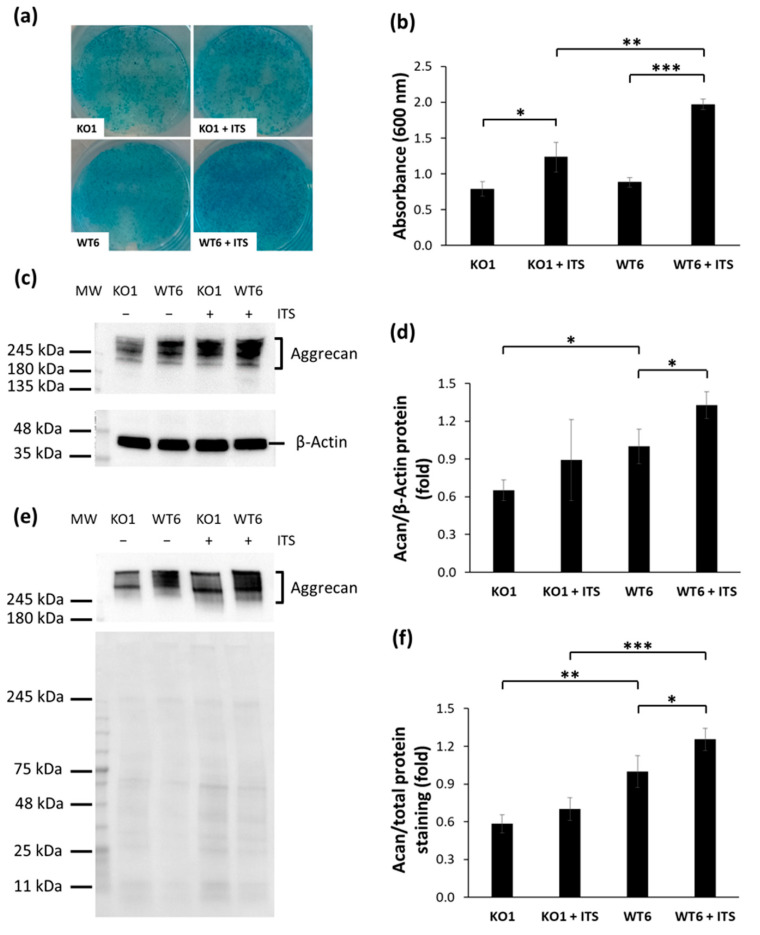
Synthesis and deposition of extracellular matrix proteins in immortalized chondrocytes. (**a**) Alcian blue staining of immortalized chondrocytes incubated with or without ITS for 12 days. (**b**) Spectrophotometric measurement after Alcian blue extraction. Data are reported as mean ± standard deviation of three replicates. Significance among samples was analyzed by Student’s *t*-test; * *p* < 0.05; ** *p* < 0.01; *** *p* < 0.001. Alcian blue staining demonstrated that after 12 days of ITS incubation PG deposition was increased in both immortalized *Cant1* knock-out and wild-type chondrocytes and WT6 cells synthesized more PGs compared with KO1. (**c**) Western blot of proteins extracted from the cell layer of immortalized chondrocytes incubated with or without ITS for 12 days was performed with specific antibodies against aggrecan core protein and against β-Actin as internal control. MW = molecular weight. (**d**) Densitometric quantification of blots from cell layer proteins. The aggrecan band was normalized to the β-Actin band. The intensity of WT6 band was set to one and the expression of other samples was expressed as fold change. Data are reported as mean ± standard deviation of three independent experiments. Significance among samples was analyzed by Student’s *t*-test; * *p* < 0.05. (**e**) Western blot of proteins from the medium of immortalized chondrocytes incubated with or without ITS for 12 days was performed with an antibody against the aggrecan core protein; the protein stain of the PVDF membrane with swift membrane stain was used as loading control. MW = molecular weight. (**f**) Densitometric quantification of blots from medium proteins. The aggrecan band was normalized to total protein staining of the PVDF membrane. The intensity of WT6 band was set to one and the expression of other samples was expressed as fold change. Data are reported as mean ± standard deviation of four independent experiments. Significance among samples was analyzed by Student’s *t*-test; * *p* < 0.05; ** *p* < 0.01; *** *p* < 0.001. Western blot of cell layer and medium proteins showed increased aggrecan synthesis in WT6 chondrocytes after ITS incubation and a higher aggrecan synthesis in WT6 cell line compared with immortalized KO1 chondrocytes. The full original Western blots are reported in Supplementary Materials Figure S2.

Alcian blue staining, a cationic dye that stains sulfated GAGs, confirmed the deposition of sulfated PGs in the ECM in both cell lines either in presence or absence of ITS. When ITS was present, PG deposition in the cell layer was significantly increased demonstrating that both cell lines were responsive to ITS (*p* = 0.028 and *p* = 0.00005 in KO1 and WT6, respectively) (Figure 5a,b). Moreover, after ITS supplementation, PG deposition in the ECM was higher in WT6 compared with KO1 (1.970 ± 0.07 vs. 1.233 ± 0.20 absorbance units, respectively; *p* = 0.004) (Figure 5b) confirming the reduced capability of immortalized *Cant1* knock-out chondrocytes to synthesize ECM macromolecules. 

PG deposition in the cell layer was further studied by aggrecan Western blots (Figure 5c). Densitometric analysis demonstrated that aggrecan in the ECM was slightly increased when the KO1 cell line was incubated with ITS, while after ITS supplementation WT6 cell line showed a significant increase of aggrecan (*p* = 0.03). When cells were incubated in basal medium a significantly higher amount of aggrecan was detected in WT6 compared with KO1, while after ITS incubation WT6 showed an increase in aggrecan synthesis compared with KO1, even if not statistically relevant (Figure 5d). To further characterize aggrecan synthesis, the 24 h culture medium at the end of 12 days ITS incubation was analyzed by Western blot (Figure 5e). Aggrecan synthesis was increased after incubation with ITS in both cell lines, even if it was statistically significant only in WT6 cell line (*p* = 0.017). Either in the presence or in absence of ITS, aggrecan synthesis was significantly higher in WT6 compared with KO1 (*p* = 0.0001 and *p* = 0.0013 in presence and in absence of ITS, respectively) (Figure 5f).

**Figure 6:**
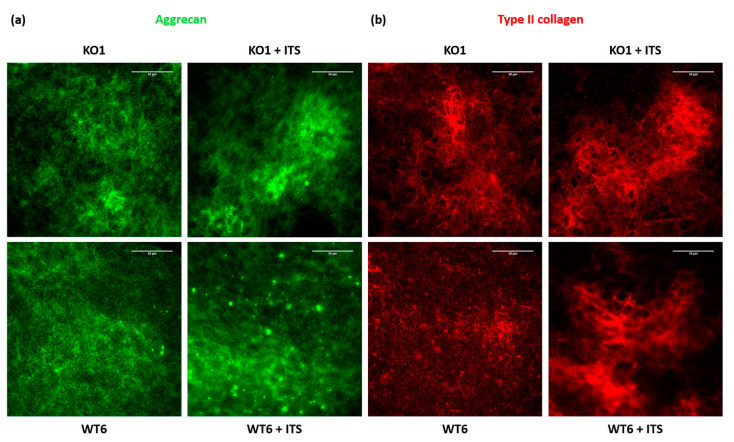
Immunofluorescence study of the extracellular matrix produced by immortalized chondrocytes incubated with or without ITS for 12 days. (**a**) Immunofluorescence analysis with a specific antibody against aggrecan. (**b**) Immunofluorescence analysis with a specific antibody against type II collagen. Both aggrecan and type II collagen showed an increased deposition and a better-defined network in immortalized chondrocytes, both *Cant1* knock-out (KO1) and wild-type (WT6), after incubation with ITS. Scale bar 50 µm.

The ECM produced after 12 days in presence or absence of ITS was also studied by immunofluorescence analysis with specific antibodies against aggrecan and type II collagen. Increased matrix deposition of both aggrecan and type II collagen was observed in immortalized *Cant1* knock-out and wild-type chondrocytes incubated with ITS compared with immortalized cells incubated in DMEM with 5% FBS. Moreover, in immortalized cells incubated with ITS a better-defined network of aggrecan and type II collagen was observed compared with cells grown in basal medium (Figure 6).

In conclusion, these data demonstrated that ECM is deposited in long term culture of immortalized chondrocytes, in particular when ITS was present in the culture medium. Moreover, immortalized KO1 chondrocytes showed a reduced deposition of ECM molecules compared with immortalized WT6 cells confirming the GAG synthesis defect described above and previously demonstrated in *Cant1* knock-out primary chondrocytes [33].

## 3. Discussion

Chondrocytes are the sole cell type present in cartilage tissue; thus, they are the central player in the pathophysiology of cartilage disorders. Therefore, chondrocytes have been widely used to study the molecular and cellular regulation of the cells and their role in cartilage metabolism. However prolonged time in culture leads to the de-differentiation of chondrocytes characterized by loss of chondrogenic phenotype and decreased expression of ECM genes limiting their use in research [36]. To overcome these drawbacks chondrocyte cell lines with the ability to maintain or re-express their phenotype have been developed using different approaches: re-differentiation using 3D gels, establishment of chondrosarcoma cell lines and immortalization with viral infection or plasmid transfection [13,14,15,16].

The immortalized chondrocytes described so far include normal cells from different species that have been immortalized as an additional tool to study specific aspect of chondrocyte biology in normal condition or in osteoarthritis, the most common degenerative disorder of articular cartilage [25,26,27]. The functions of specific cartilage macromolecules have been studied also in vivo using different murine models of osteoarthritis as well as animal models of genetic disorders of the skeleton [6,8]. Cell lines established from genetically modified animals would also play important roles in the detailed understanding of the cartilage defects reflecting the genetic aberration and would ultimately reduce the use of transgenic animals for primary cells according to the principle of the Three Rs. In such investigations, cell lines that accurately reproduce the characteristics of primary cells in culture are important and can serve as appropriate model systems. In the last several years, in vitro testing has served as the primary testing model for drugs because it helps bringing new drugs to in vivo studies and then to clinical trials faster and with reduced costs [37]. However, there is a caveat: The in vitro preclinical tools should require a high amount of well-characterized cells, a condition that cannot be achieved with primary chondrocytes, but may be satisfied using immortalized chondrocyte cell lines.

In this work, we successfully established immortalized chondrocytes from *Cant1* knock-out and wild-type mice using a plasmid vector expressing the SV40 large and small T antigen which have been used for human, murine, and rabbit chondrocyte immortalization [17,18,21,26,27,38]. Our results demonstrated the generation of stable cell lines of immortalized murine chondrocytes that express the differentiated phenotype. Proliferation in monolayer culture in the presence of 10% FBS was quite fast and immortalized cells showed a morphology similar to primary chondrocytes. Since immortalization produces a continuous state of proliferation it is important to consider the maintenance of the chondrocyte phenotype. To assess the chondrocyte phenotype, the cell lines were characterized for type II collagen and aggrecan expression at the protein and mRNA level. Among the clones, KO1 and WT6 cell lines showed significantly higher type II collagen and aggrecan expression compared with other clones and for this reason they were further characterized. These observations suggest that SV40 large T antigen expression maintain chondrocytes in a proliferative state preserving the cartilage phenotype. 

We previously demonstrated that *Cant1*, a calcium-activated nucleotidase of the Golgi, is important for GAG synthesis [33,35]. Mutations in this gene cause DBQD1 a skeletal disorder belonging to the multiple dislocation group and characterized by severe prenatal and postnatal growth retardation, joint laxity, short extremities, and progressive scoliosis [34]. The functional impairment of the enzyme causes reduced GAG synthesis, chondroitin sulfate oversulfation, reduced hydrodynamic size of GAGs and delayed PG secretion as demonstrated in primary chondrocyte cultures [33]. Thus, once we selected the immortalized cell lines with the chondrocyte phenotype, we checked whether immortalized *Cant1* knock-out cells maintain the phenotypic defects observed in primary chondrocytes. After metabolic labeling with ^35^S-sulfate of immortalized cell lines, we observed reduced GAG synthesis either in basal medium or in medium containing *p*-nitrophenyl β-D-xylopyranoside in immortalized *Cant1* knock-out chondrocytes compared with immortalized wild-type cells. As already observed in primary culture, the defect was more enhanced in presence of *p*-nitrophenyl β-D-xylopyranoside, a molecule acting as a chain initiator for GAG synthesis. Furthermore, consistent with what observed in primary *Cant1* knock-out cells, chondroitin sulfate GAGs were oversulfated in immortalized *Cant1* knock-out chondrocytes compared with wild-type. Overall, these results confirmed the GAG synthesis defect in immortalized *Cant1* knock-out cells as already observed in primary cells validating the in vitro model.

Immortalized human chondrocytes have been proposed as a good surrogate model to study specific feature of chondrocytes by Hoffman et al.; they demonstrated that clones with a chondrocyte phenotype showed gene expression modulation to an anabolic and catabolic factor, BMP-7 and IL-1, respectively, similar to primary chondrocytes [27]. Correspondingly immortalized mouse chondrocytes either in monolayer or in alginate stimulated with BMP-2 resulted in increased expression of ECM molecules such as type II collagen and aggrecan, while treatment with the pro-inflammatory IL-1 caused increased gene expression of catabolic effectors mimicking primary chondrocytes [26]. 

PG production by immortalized human chondrocyte cell lines have been reported both in monolayer and alginate cultures since aggrecan, the large aggregating PG of cartilage, is not only a marker of differentiation, but also an essential constituent of the cartilage ECM. It has been reported that in T/C-28a2 and tsT/AC62 cell lines the expression of aggrecan is higher in monolayer compared with alginate cultures. Moreover, since alginate did not expand or remodel, clusters of cells were formed and this might affect cell survival [21]. In the same work it has been reported that FBS favors PG synthesis indicating that the growth factors present in serum were important for ECM synthesis. The capacity of insulin to stimulate PG synthesis has been well documented [18,39,40]. Furthermore, ITS, a serum substitute containing insulin, transferrin, and sodium selenite, increases ECM deposition in long-term cultures as already observed in ATDC5 cells [28]. For this reason, the ECM produced by immortalized cells after long term culture (12 days) either in presence or absence of ITS was investigated by Alcian blue staining, Western blot, and immunofluorescence studies. All experiments demonstrated that in both cell lines ITS favored ECM synthesis suggesting its use to deeply study the synthesis and deposition of ECM molecules using immortalized chondrocytes. Moreover, the ECM deposition was higher in the wild-type compared with the *Cant1* knock-out cell line confirming the GAG synthesis defect in immortalized *Cant1* knock-out chondrocytes. Interestingly, these data demonstrated that a denser matrix was present in immortalized wild-type chondrocytes compared with immortalized *Cant1* knock-out cells as previously observed ex vivo by TEM studies in cartilage from the *Cant1* knock-out mouse [33].

In conclusion, we immortalized chondrocytes from our mouse model of DBQD1 and we demonstrated that the phenotype of immortalized cells was similar to the one of primary chondrocytes based on PG synthesis, sulfation, and ECM deposition. This cell line is a useful culture model for studying specific steps of PG synthesis, a complex and not well-characterized process. Moreover, since PGs are important components of the cartilage ECM, the model should provide additional information on how the defect in PG biosynthesis could affect the organization of the ECM. This successful immortalization procedure could be applied to cells from other animal models to increase the tools available for physiopathological studies and drug discovery. 

## 4. Materials and Methods

### 4.1. Animal Model and Care

In this study, wild-type and homozygous *Cant1* knock-out mice with a C57Bl/6J × 129/SV background were used [33]. Animals were genotyped by polymerase chain reaction (PCR) using genomic DNA extracted from mouse tail clips.

Care and use of mice complied with relevant animal welfare institutional guidelines and protocols were approved by the Animal Care and Use Committee of the University of Pavia and the Ministry of Health (Licence n. 486/2019-PR released on 4 July 2019). 

### 4.2. Chondrocyte Isolation and Immortalization

To establish primary chondrocyte cultures, femoral head cartilage of four-day-old mice was harvested and digested with 2 mg/mL of collagenase type II (Invitrogen, Thermo Fisher, Waltham, MA, USA) in Dulbecco’s modified Eagle’s medium (DMEM; Sigma–Aldrich, Milan, Italy) at 37 °C overnight. Released chondrocytes were plated and cultured in DMEM with 10% fetal bovine serum (FBS; EuroClone, Milan, Italy) at 37 °C in a humidified atmosphere containing 5% CO_2_. 

The day before transfection, primary chondrocytes were plated in a 24-well plate at 60 kcell/well in 1 mL of DMEM with 10% FBS. Cells were transfected in 0.5 mL of DMEM containing 10% FBS with the pZipSV766-1 plasmid [17,41,42] (kindly provided by Dr. Adriana Zingone, National Cancer Institute, Bethesda, USA) expressing the large and small T antigen of SV40 using the Lipofectamine^®^ LTX & PLUS™ Reagent (Invitrogen, Thermo Fisher, Waltham, MA, USA) in accordance with the manufacturer’s instruction. For stable transfection, two days after transfection cells were selected with 0.25 mg/mL G418 (Roche, Mannheim, Germany) in DMEM with 10% FBS for two weeks changing the medium every two days. Then cells were passaged to 10-cm^2^ Petri dishes and the selection with 0.25 mg/mL G418 was maintained for another two weeks. For clonal isolation, diluted cells were plated in 10-cm^2^ Petri dishes and clones were transferred to 96-well plates and on confluence expanded sequentially to culture dishes of increased cell culture area. Images of cells were acquired with a Leica DMIL LED microscope (Leica, Milan, Italy) connected to a Leica DFC480 camera at 20× magnification using the LAS 3.0.0 software (Leica, Milan, Italy). For all the experiments, cells were used from passage 10 to 20.

### 4.3. Collagen Analysis

Immortalized cells in a 75 cm^2^ flask were incubated in DMEM without FBS supplemented with 100 µg/mL sodium ascorbate at 37 °C in 5% CO_2_ for 48 h. After incubation, the medium was harvested, and protease inhibitors (10 mM of benzamidine, 2 mM of N-ethylmaleimide (NEM), 4 mM of ethylenediaminetetraacetic acid (EDTA), 1 mM of phenylmethylsulfonyl fluoride (PMSF) final concentration) were added. Then proteins were precipitated with 176 mg/mL of ammonium sulfate overnight at 4 °C. Collagens were purified by digestion with 100 µg/mL of pepsin (Sigma–Aldrich, Milan, Italy) in 0.5 M of acetic acid, pH 2.0, followed by precipitation with 2 M of NaCl in 0.5 M of acetic acid. Collagens were denatured in Laemmli buffer (62.5 mM of Tris-HCl, pH 6.8, 10% glycerol, 2% sodium dodecyl sulphate (SDS), 0.01% bromophenol blue) and analyzed by SDS-PAGE on 6% polyacrylamide gels in non-reducing condition. Gels were stained with Coomassie Brilliant Blue R-250 (Bio-Rad, Milan, Italy). Images were acquired using Chemidoc XRS apparatus (Bio-Rad, Milan, Italy). Densitometry analysis was performed using the ImageQuant TL v8.1.0.0 software (GE Healthcare Bio-Sciences, Piscataway, NJ, USA). 

### 4.4. Real-Time PCR

Total RNA was extracted from a 10-cm^2^ Petri dish of confluent immortalized chondrocytes by QIAzol^®^ Lysis Reagent and miRNeasy Mini Kit (QIAGEN, Milan, Italy) in accordance with the manufacturer’s instructions. Genomic DNA was removed by DNA digestion using RNase-Free DNase Set (QIAGEN, Milan, Italy). Then cDNA was obtained from 1 µg of purified RNA by SuperScript^TM^ IV First-Strand Synthesis System (Invitrogen, Thermo Fisher, Waltham, MA, USA) in accordance with the manufacturer’s protocol. 

Quantitative real-time PCR experiments were performed using the QuantiFast SYBR Green PCR Kit (QIAGEN, Milan, Italy) with QuantiTect Primer Assay (QIAGEN, Milan, Italy) for type II collagen (Col2a1, QT01055523), aggrecan (Acan, QT00175364), and glyceraldehyde 3-phosphate dehydrogenase (Gapdh, QT01658692) as a housekeeping gene for expression normalization. Each sample was run in triplicate in 96-well plates with the QuantStudio 3 (Applied Biosystems, Thermo Fisher, Waltham, MA, USA) apparatus and relative gene expression was determined with the ΔΔCt method.

### 4.5. Analysis of Proteoglycan Synthesis by Metabolic Labeling

Immortalized chondrocytes were plated in six-well plates at 250 kcell/well in DMEM containing 10% FBS. After 48 h, cells were preincubated with or without 1 mM *p*-nitrophenyl β-d-xylopyranoside (Sigma–Aldrich, Milan, Italy) in minimal essential medium (MEM; Sigma–Aldrich, Milan, Italy) containing 250 µM cold Na_2_SO_4_ without FBS at 37 °C in 5% CO_2_ for two hours. Then cells were labeled with 50 µCi/mL Na_2_[^35^SO_4_] (38.8-59.2 TBq/mmol, PerkinElmer, Waltham, MA, USA) in the same medium for 24 h as previously described [43]. Briefly, the medium was harvested with an equal volume of 100 mM sodium acetate buffer, pH 5.8, containing 8 M urea, 4% Triton X-100, 20 mM EDTA, 20 mM NEM, 0.1 M 6-aminocaproic acid and 1 mM PMSF, while the cell layer was lysed in 50 mM sodium acetate buffer, pH 5.8, containing 2 M urea and 2% Triton X-100. An aliquot of cell lysates was used to determine the protein content by the BCA Protein Assay (Thermo Fisher, Waltham, MA, USA) and the rest was added to the medium. PGs were then purified by DEAE Sephacel chromatography (GE Healthcare Bio-Sciences, Piscataway, NJ, USA) as previously described [33]. Then, PGs were quantified by measuring the ^35^S-activity using a liquid scintillation counter (TRI-CARB 2300 TR, PerkinElmer, Waltham, MA, USA) and normalized to the protein content. 

### 4.6. Proteoglycan Sulfation Analysis

For PG sulfation analysis, immortalized chondrocytes in a 25 cm^2^ flask were incubated with DMEM without FBS at 37 °C in 5% CO_2_ for 24 h. The medium was made 0.1 M of sodium acetate, pH 5.6, 5 mM of EDTA, and 5 mM of cysteine, and digested with 20 U of papain (Sigma–Aldrich, Milan, Italy) at 65 °C overnight. Then papain was inactivated at 100 °C for 10 min and released GAGs were recovered by cetylpyridinium chloride precipitation as previously described [33]. Recovered GAGs were digested with 20 mU of chondroitinase ABC (AMSBIO, Abingdon, UK) and 20 mU of chondroitinase ACII (Sigma–Aldrich, Milan, Italy) in 0.1 M of ammonium acetate buffer, pH 7.35, and released chondroitin sulfate disaccharides analyzed by HPLC after 2-aminoacridone (Invitrogen, Thermo Fisher, Waltham, MA, USA) derivatization as previously described [44,45].

### 4.7. Western Blot Analysis

For Western blot analysis, 1.5 × 10^6^ immortalized chondrocytes were plated in 60-mm-diameter culture dishes with DMEM and 10% FBS and incubated at 37 °C in 5% CO_2_. After three days, cells were incubated in DMEM without FBS at 37 °C in 5% CO_2_ for 24 h. For insulin–transferrin–selenium (ITS) incubation, 4.4 × 10^5^ immortalized chondrocytes were plated in 60 mm culture dishes with DMEM and 10% FBS and incubated at 37 °C in 5% CO_2_. When cells reached 50–70% confluency, the medium was changed with DMEM and 5% FBS with or without ITS (Gibco, Thermo Fisher, Waltham, MA, USA). The medium was changed every two or three days. After 12 days of ITS incubation, cells were incubated in DMEM without FBS at 37 °C in 5% CO_2_ for 24 h. At the end of the incubation period, the medium was harvested, and the cell layer was scraped in PBS, centrifuged, and the pellets were lysed in RIPA buffer. Medium and lysed cells were ultrafiltered with Amicon Ultra centrifugal filter units (10 kDa cut-off, Merck Millipore, Milan, Italy) in 0.1 M of ammonium acetate, pH 7.35, and lyophilized. The lyophilizate was dissolved in 200 µL 0.1 M ammonium acetate, pH 7.35, and an aliquot of the cell layer fraction was used for protein quantitation by BCA Protein Assay (Thermo Fisher, Waltham, MA, USA). All samples were digested with 40 mU of chondroitinase ABC (Seikagaku Corporation, Tokyo, Japan) at 37 °C overnight to remove GAGs and unmask the epitope on the aggrecan core protein. Then samples were lyophilized and resuspended in Laemmli buffer. SDS-PAGE was performed on 4–15% polyacrylamide gradient gels (Bio-Rad, Milan, Italy) and proteins were transferred on polyvinylidene difluoride (PVDF) membrane (Amersham Biosciences, Amersham, UK). Membranes were blocked with 5% bovine serum albumin (BSA; Sigma–Aldrich, Milan, Italy) 0.05% Tween-20 (Sigma–Aldrich, Milan, Italy) in Tris-buffered saline (TBS), and incubated with primary antibodies against aggrecan (1:500, rabbit monoclonal antibody, Merck Millipore, Milan, Italy), β-actin (1:5000 mouse monoclonal antibody, Merck Millipore, Milan, Italy), and the appropriate HRP secondary antibody (1:2000, goat anti-rabbit antibody and horse anti-mouse antibody, Cell Signaling, Danvers, MA, USA). Aggrecan and β-actin bands were detected by Westar η C 2.0 (Cyanagen, Bologna, Italy) and images were acquired by ImageQuant LAS 4000 (GE Healthcare Bio-Sciences, Piscataway, NJ, USA). The ImageQuant TL v8.1.0.0 software (GE, Healthcare Bio-Sciences, Piscataway, NJ, USA) was used for densitometry analysis. For cell layer samples, the intensity of the aggrecan band was normalized to the intensity of the β-actin band as a loading control. For normalization of medium samples, before the blocking step, the membrane was stained with the swift membrane stain (G-Biosciences, St Louis, MO, USA) in accordance with the manufacturer’s instructions and scanned with ImageQuant LAS 4000 (GE, Healthcare Bio-Sciences, Piscataway, NJ, USA); the intensity of the aggrecan band was normalized to the intensity of the lane after membrane staining. The intensity of the wild-type band was set to one and the expression of other samples was expressed as fold change.

### 4.8. Alcian Blue Staining

For Alcian blue staining, 40 × 10^3^ immortalized chondrocytes were plated in 24-well plates in DMEM and 10% FBS and incubated at 37 °C in 5% CO_2_. When cells reached 50–70% confluency, the medium was changed with DMEM and 5% FBS with or without ITS. The medium was changed every two or three days. After 12 days of ITS incubation, chondrocytes were fixed with 10% formalin in PBS (Sigma–Aldrich, Milan, Italy) for one hour at room temperature. Then, cells were incubated with 0.5% Alcian blue 8GX (Sigma–Aldrich, Milan, Italy) in 0.1 M HCl overnight at room temperature. After one wash with 0.1 M HCl and three with distilled water, the blue dye was solubilized with 4 M guanidine HCl overnight at 4 °C and the absorbance measured with a spectrophotometer at 600 nm. 

### 4.9. Immunofluorescence Studies

To study deposition of ECM, 10 × 10^3^ immortalized chondrocytes were plated on sterile glass coverslips (Marienfeld Superior, Lauda–Königshofen, Germany) in a 24-well plate in DMEM and 10% FBS and incubated at 37 °C in 5% CO_2_. When cells reached 50–70% confluency, the medium was changed with DMEM, and 5% FBS supplemented with or without ITS. The medium was changed every two or three days. After 12 days of ITS incubation, cells were washed with PBS and the ECM was processed as previously described [46]. Briefly, samples were incubated three times for 10 min each with 0.5% sodium deoxycholate and 1 mM PMSF in 10 mM Tris-HCl, pH 8.0, at 4 °C followed by three washes 10 min each with 2 mM Tris-HCl, pH 8.0, containing 1 mM PMSF at 4 °C and fixation with 4% formaldehyde in PBS (Invitrogen, Thermo Fisher, Waltham, MA, USA) for 10 min at room temperature. Then samples were blocked with 1.5% BSA in PBS and incubated with a specific primary antibody against aggrecan (1:500, rabbit monoclonal antibody, Merck Millipore, Milan, Italy) and type II collagen (1:100, mouse monoclonal antibody, Merck Millipore, Milan, Italy) overnight at 4 °C. Then samples were incubated with appropriate secondary antibodies: anti-rabbit antibody conjugated with Alexa Fluor 488 (1:2000, Immunological Sciences, Rome, Italy) and anti-mouse antibody conjugated with Alexa Fluor 647 (1:1000, Cell Signaling, Danvers, MA, USA). Fluorescence images were acquired using a widefield Leica microscope (DM6B) driven by LasX software (Leica, Milan, Italy) and equipped with a Hamamatsu Orca-Flash 4.0 camera (Hamamatsu, Hamamatsu City, Japan) and a 63x oil immersion objective (Leica HC PL APO 63x, 1.4NA) (Leica, Milan, Italy).

### 4.10. Statistical Analysis

Statistical analysis was performed by Microsoft Excel software. All values are reported as mean ± standard deviation. Statistical difference between groups was evaluated using Student’s *t*-test and a *p*-value < 0.05 was considered statistically significant.

## Data Availability

Data are available within the article or Supplementary Materials.

## Supplementary Materials

Supplementary materials are available online at https://www.mdpi.com/article/10.3390/ijms22179304/s1.

## Abbreviations

Acan	aggrecan
BCA	bicinchoninic acid
BMP	bone morphogenetic protein
BSA	bovine serum albumin
CANT1	calcium activated nucleotidase 1
Col2a1	α1 chain of type II collagen
COMP	cartilage oligomeric matrix protein
DBQD1	Desbuquois dysplasia type 1
DMEM	Dulbecco’s modified Eagle’s medium
ECM	extracellular matrix
EDTA	ethylenediaminetetraacetic acid
ER	endoplasmic reticulum
FBS	fetal bovine serum
GAG	glycosaminoglycan
Gapdh	glyceraldehyde 3-phosphate dehydrogenase
HPLC	high performance liquid chromatography
IL-1	interleukin-1
ITS	insulin-transferrin-selenium
MEM	minimal essential medium
NEM	N-ethylmaleimide
PBS	phosphate buffered saline
PCR	polymerase chain reaction
PG	proteoglycan
PMSF	phenylmethylsulfonyl fluoride
PVDF	polyvinylidene difluoride
SDS	sodium dodecyl sulfate
SDS-PAGE	sodium dodecyl sulfate-polyacrylamide gel electrophoresis
TBS	Tris buffered saline
TEM	transmission electron microscopy
ΔDi-0S	3-O-β(D-gluc-4-eneuronosyl)-N-acetylgalactosamine
ΔDi-4S and ΔDi-6S	derivatives of ΔDi-0S with a sulfate at the 4 or 6 position of hexosamine moiety respectively
