# Supplementary material for: Phenotypic Characterization of Immortalized Chondrocytes from a Desbuquois Dysplasia Type 1 Mouse Model: A Tool for Studying Defects in Glycosaminoglycan Biosynthesis
Source: Int J Mol Sci. 2021 Aug 27;22(17):9304. doi: 10.3390/ijms22179304 (PMC8431031; doi:10.3390/ijms22179304)
Supplement: Supplementary file 1 [file ijms-22-09304-s001.zip › ijms-1293483-supplementary.pdf]

Supplementary Figure S1

(a)

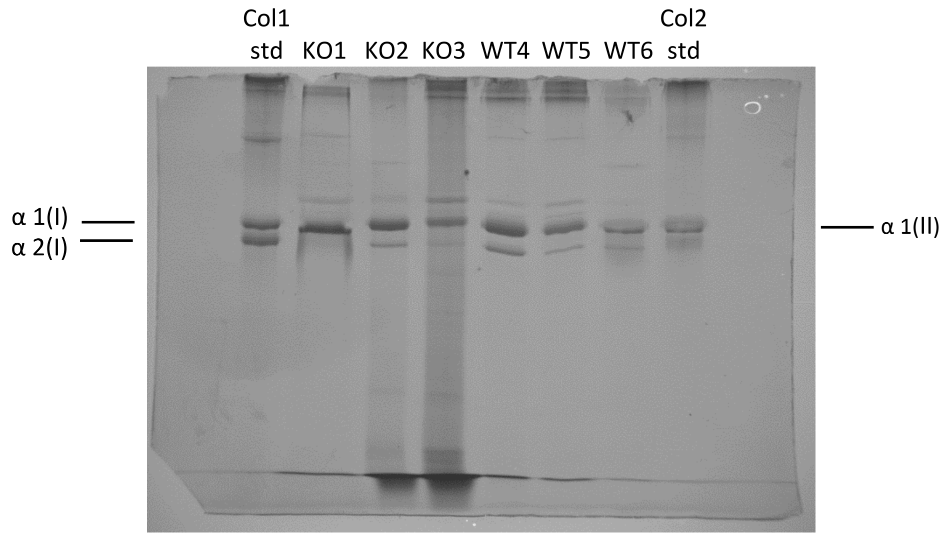

(b)

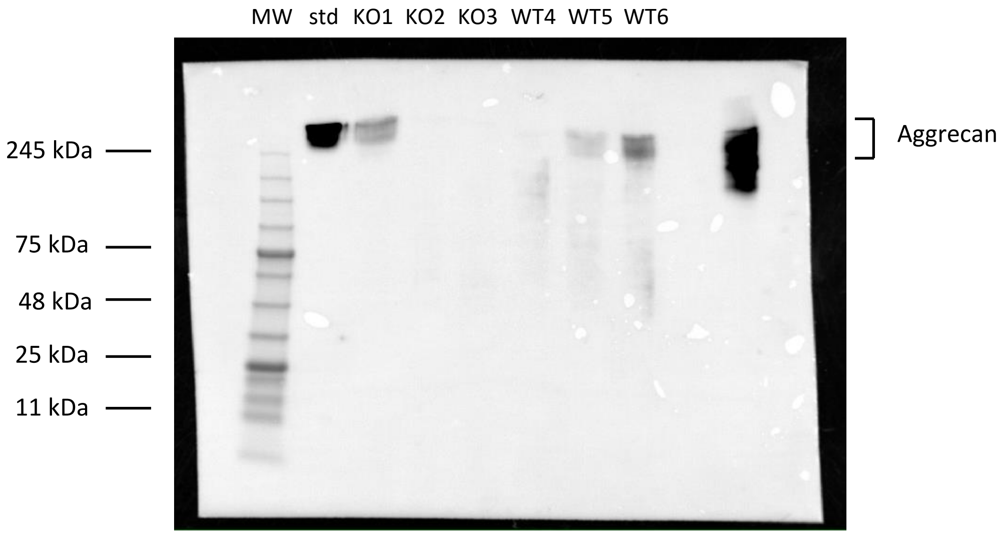

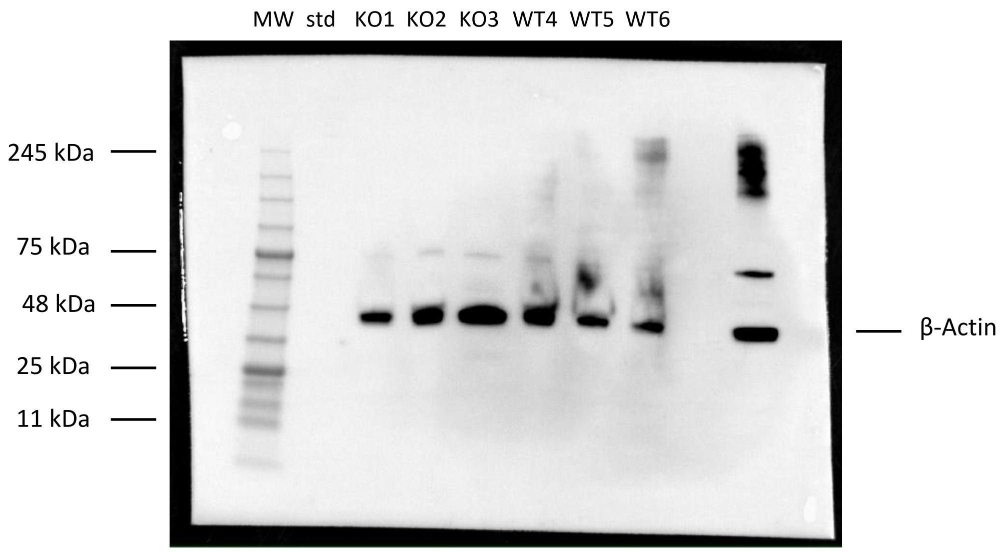

(c)

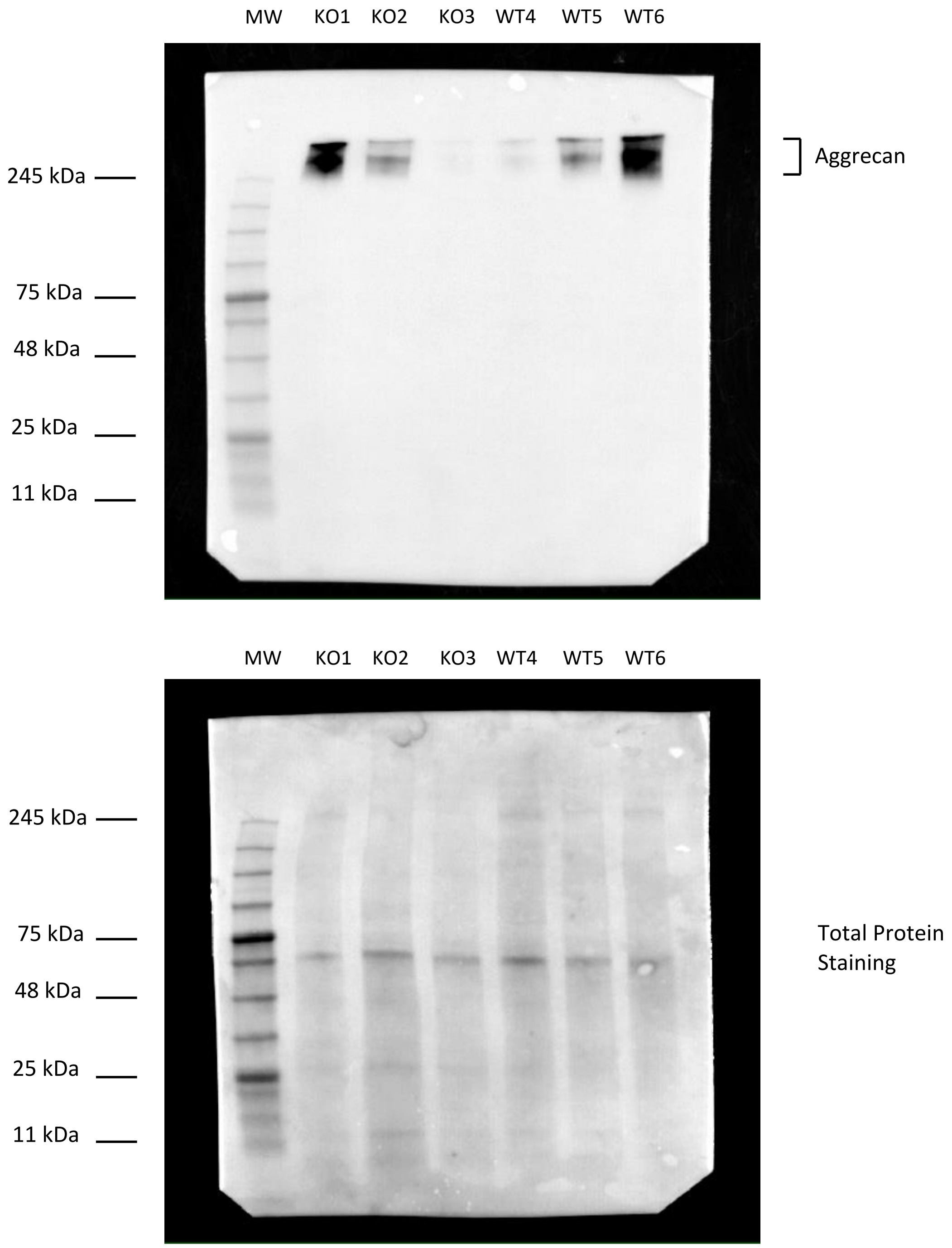

Supplementary Figure S1: Original gel and Western blots of Figure 2. (**a**) Original full size 6% SDS-PAGE of collagen extracted from medium of immortalized chondrocytes and digested by pepsin as described in Figure 2a. (**b**) Original full size of Western blot of proteins extracted from cell layer of immortalized chondrocytes and incubated with specific antibodies against aggrecan and β-Actin as described in Figure 2b. (**c**) Original full size of Western blot of proteins extracted from medium of immortalized chondrocytes and incubated with specific antibody against aggrecan and original full size of PVDF membrane after total protein staining by swift membrane stain as described in Figure 2c.

Supplementary Figure S2

(a)

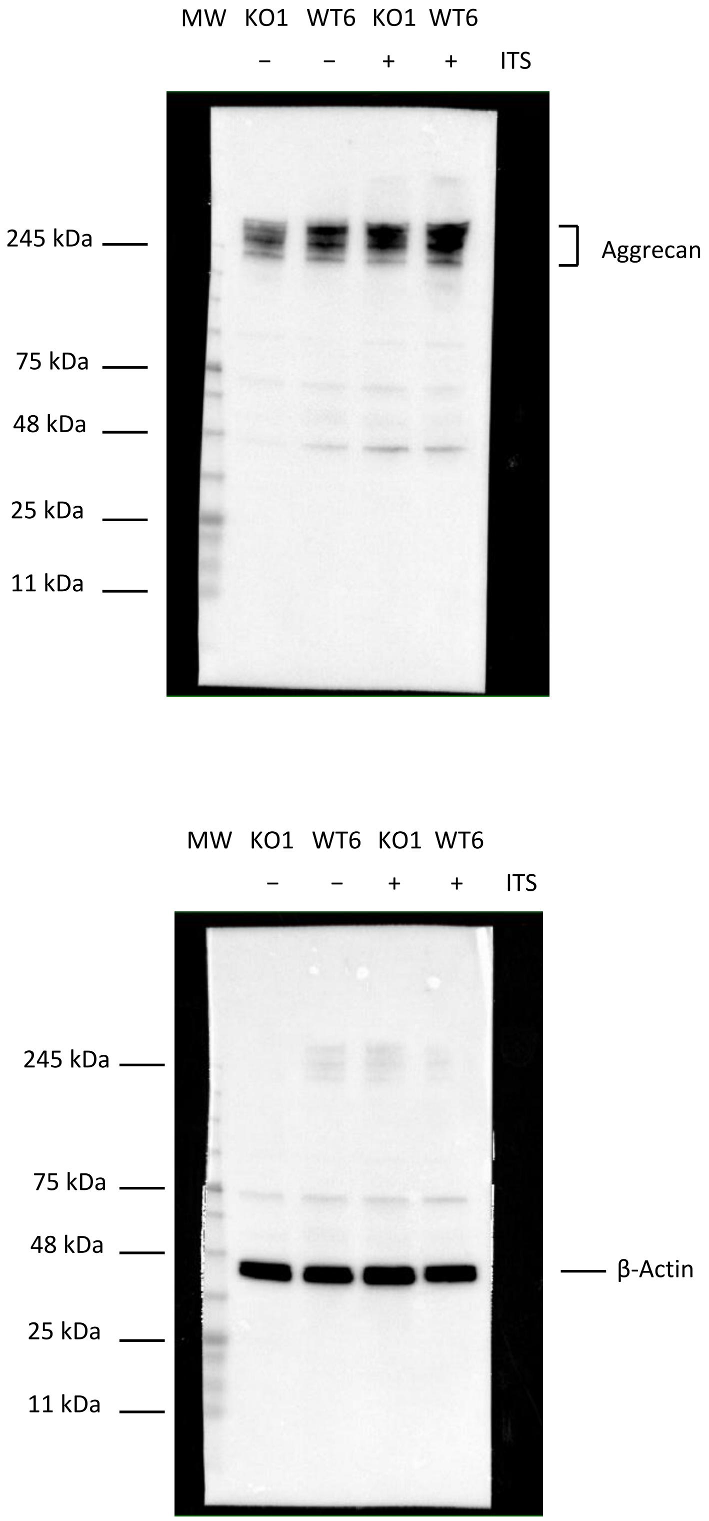

(b)

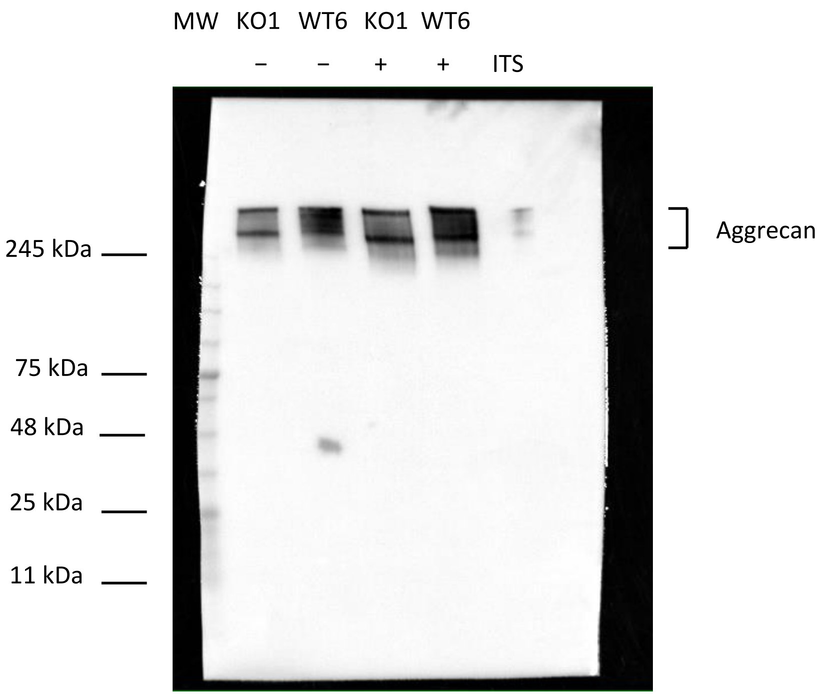

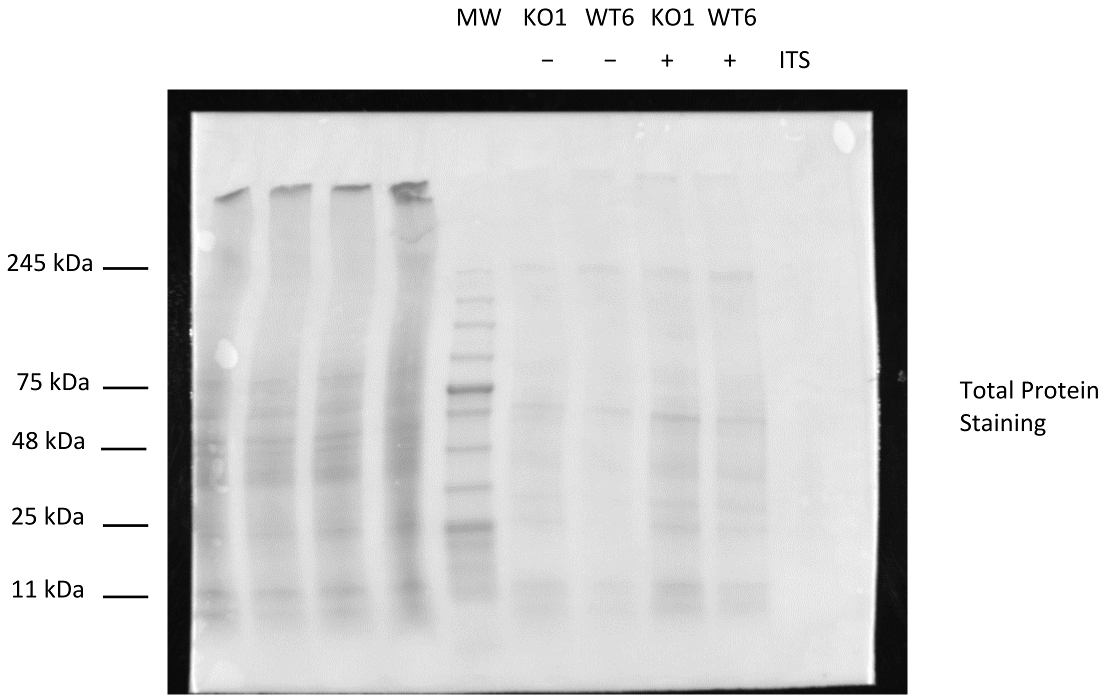

Supplementary Figure S2: Original Western blots of Figure 5. (**a**) Original full size of Western blot of proteins extracted from cell layer of immortalized chondrocytes and incubated with specific antibodies against aggrecan and β-Actin as described in Figure 5c. (**b**) Original full size of Western blot of proteins extracted from medium of immortalized chondrocytes and incubated with specific antibody against aggrecan and original full size of PVDF membrane after total protein staining by swift membrane stain as described in Figure 5e.
